# Impact of thyroid immune-related adverse events on clinical outcomes in non-small cell lung cancer (NSCLC) patients treated with checkpoint inhibitor therapy: A single center study

**DOI:** 10.17305/bb.2025.12321

**Published:** 2025-03-24

**Authors:** Šejla Cerić, Timur Cerić, Emir Sokolović, Jasmina Dalač, Dragana Miletić, Inga Marijanović, Layan Mattar, Amina Aljić, Selma Agić-Bilalagić, Amera Šadija, Miran Hadžiahmetović, Semir Bešlija

**Affiliations:** 1 Clinic for Nuclear Medicine, Clinical Center University of Sarajevo, Sarajevo, Bosnia and Herzegovina; 2 Clinic of Oncology, Clinical Center University of Sarajevo, Sarajevo, Bosnia and Herzegovina; 3 Department for Nuclear Medicine, General Hospital “Prim. Dr. Abdulah Nakaš” Sarajevo, Bosnia and Herzegovina; 4 Clinic of Oncology, University Clinical Hospital, Mostar, Bosnia and Herzegovina; 5 Faculty of Medicine, University of Sarajevo, Sarajevo, Bosnia and Herzegovina

**Keywords:** Non-small cell lung cancer, NSCLC, immunotherapy, immune checkpoint inhibitors, ICIs, thyroid dysfunction, immune-related adverse events, irAEs, atezolizumab, pembrolizumab, progression-free survival, PFS

## Abstract

Immune checkpoint inhibitors (ICIs) have transformed the treatment landscape for non-small cell lung carcinoma but are associated with immune-related adverse events (irAEs), including thyroid dysfunction. This study examines the incidence and clinical impact of thyroid dysfunction in non-small cell lung cancer (NSCLC) patients receiving ICIs at the Clinic of Oncology, Clinical Center University of Sarajevo. In this retrospective cohort study of 50 patients with metastatic NSCLC treated with ICIs—either in combination with chemotherapy or as monotherapy for those with programmed death-ligand 1 (PD-L1) expression ≥ 50%—we collected data on demographics, treatment regimens, thyroid function tests, and survival outcomes. Thyroid dysfunction occurred in 24 patients (48%), with 12 (24%) developing hypothyroidism, 4 (8%) developing hyperthyroidism, and 8 (16%) experiencing a transition from hyperthyroidism to hypothyroidism. The incidence of thyroid dysfunction was significantly higher in patients treated with atezolizumab compared to pembrolizumab (*P* ═ 0.04), with 87.5% of affected patients receiving atezolizumab. The median time to onset of thyroid dysfunction was 10 cycles (interquartile range [IQR]: 5) for hypothyroidism and six cycles (IQR: 19) for hyperthyroidism. Progression-free survival (PFS) was significantly longer in patients who developed thyroid dysfunction, with the median PFS not reached, compared to a median PFS of 14 months (95% CI: 9.68–18.32) in patients without thyroid dysfunction (*P* ═ 0.038). No significant associations were found between thyroid dysfunction and patient age or gender. These findings suggest that thyroid dysfunction is a common irAE in patients with metastatic NSCLC receiving ICIs, particularly atezolizumab, and its development may be associated with improved PFS. Regular monitoring of thyroid function is recommended to promptly identify and manage thyroid abnormalities during ICI therapy, potentially improving patient outcomes.

## Introduction

Non-small cell lung cancer (NSCLC) accounts for approximately 85% of all lung cancer cases and remains a leading cause of cancer-related mortality worldwide [1]. Despite advances in treatment, the prognosis for patients with metastatic NSCLC remains poor, highlighting the urgent need for more effective therapies [2].

The introduction of immune checkpoint inhibitors (ICIs) has transformed the treatment landscape of NSCLC. Agents, such as pembrolizumab and atezolizumab work by blocking inhibitory pathways—specifically programmed cell death protein 1 (PD-1) and programmed death-ligand 1 (PD-L1)—thereby enhancing T-cell–mediated anti-tumor responses [3, 4]. Clinical trials have shown that ICIs improve survival outcomes in patients with advanced NSCLC, either as monotherapy or in combination with chemotherapy, particularly among those with high PD-L1 expression [5–7].

However, by activating the immune system, ICIs can cause immune-related adverse events (irAEs), which may affect any organ system. Among the most common are endocrine irAEs, with thyroid dysfunction being particularly prevalent [8, 9]. Thyroid dysfunction typically presents as either hypothyroidism or hyperthyroidism and can significantly affect a patient’s quality of life and adherence to treatment if not promptly recognized and managed [10].

The pathogenesis of ICI-induced thyroid dysfunction is not yet fully understood. Proposed mechanisms include immune-mediated destruction of thyroid cells, the induction of thyroid autoantibodies, and cross-reactivity between tumor antigens and thyroid tissue [10–12]. A genetic predisposition may also contribute, as suggested by associations with specific human leukocyte antigen (HLA) genotypes [13].

Interestingly, emerging evidence suggests that the development of irAEs, including thyroid dysfunction, may be associated with improved clinical outcomes in patients receiving ICIs [14, 15]. This correlation raises the possibility that thyroid irAEs could serve as a biomarker for treatment efficacy, although current findings are inconsistent and warrant further investigation.

Despite the clinical significance of thyroid dysfunction during ICI therapy, data on its incidence and impact in patients with NSCLC—particularly in relation to specific ICIs, such as pembrolizumab and atezolizumab—remain limited. A clearer understanding of the incidence, timing, and clinical implications of thyroid irAEs is essential for optimizing patient management, improving outcomes, and guiding monitoring strategies during treatment.

This study aims to investigate the incidence and clinical impact of thyroid dysfunction in patients with metastatic NSCLC treated with ICIs at the Clinic of Oncology, Clinical Center University of Sarajevo. By examining the association between thyroid dysfunction and clinical outcomes, such as progression-free survival (PFS), the research seeks to improve understanding of thyroid irAEs in this patient population and contribute to the development of evidence-based management guidelines.

## Materials and methods

### Study design and setting

This retrospective cohort study was conducted at the Clinic of Oncology, Clinical Center University of Sarajevo, a tertiary care hospital serving a diverse patient population in Bosnia and Herzegovina. We retrospectively identified all patients who initiated ICI therapy at our institution between January 2020 and January 2022. Data collection continued through April 2023, which served as the final analysis cutoff. During this period, the median follow-up time—from ICI initiation to last contact or censoring—was 10.6 months (interquartile range [IQR]: 4.1 months). Patients who began treatment earlier in the study period had substantially longer follow-up durations.

### Patient selection

The inclusion criteria for this study were as follows: patients had to be at least 18 years old at the time of therapy initiation and have a histologically or cytologically confirmed diagnosis of metastatic NSCLC. They must have initiated first-line ICI therapy—either as monotherapy or in combination with chemotherapy—and have available baseline and follow-up thyroid function tests (TFTs). An Eastern Cooperative Oncology Group (ECOG) performance status of 0–2 was also required for eligibility.

Exclusion criteria included any prior treatment with ICIs or participation in clinical trials involving ICIs. Patients with pre-existing thyroid dysfunction or other major endocrine disorders were deemed ineligible, as were those with concurrent malignancies or significant comorbidities that could affect survival. Lastly, individuals with incomplete medical records or those lost to follow-up before the first response evaluation were excluded from the study.

### Treatment regimens

Patients received ICIs in accordance with institutional protocols and national guidelines. Treatment options included pembrolizumab, administered as monotherapy for patients with PD-L1 expression ≥50%. Patients with PD-L1 expression <50% typically received atezolizumab, usually in combination with chemotherapy. In certain cases, treating oncologists opted for atezolizumab monotherapy based on patient comorbidities or intolerance to combination treatment. Atezolizumab was administered at a dose of 1200 mg every three weeks, while pembrolizumab was given at a dose of 200 mg every three weeks. Chemotherapy regimens, typically platinum-based doublets, were selected according to standard practice.

### Data collection

Data were extracted from electronic medical records using a standardized data collection form by trained oncology residents or attending physicians. Accuracy was ensured by cross-referencing the information with laboratory and pharmacy records.

The collected variables encompassed a broad range of patient and treatment details. Demographic data included age, gender, smoking status, and body mass index (BMI). Clinical characteristics covered histological subtype, tumor stage (TNM), PD-L1 expression level, ECOG performance status, comorbidities, and, where available, tumor grade. Treatment details included the type of ICI, dosing schedule, number of cycles, and any concomitant medications. Thyroid function was monitored by measuring TSH, FT4, and FT3 at baseline and prior to each treatment cycle. irAEs were documented and graded according to CTCAE v5.0. Outcomes assessed were PFS, overall survival (OS), and tumor response, as determined by RECIST v1.1.

### Definition, monitoring, and management of thyroid dysfunction

Thyroid dysfunction was diagnosed based on TFTs and clinical evaluation. Hypothyroidism was defined as a TSH level >4.0 mIU/L with either decreased or normal FT4, while hyperthyroidism (thyrotoxicosis) was defined as a TSH level <0.4 mIU/L accompanied by elevated FT4 and/or FT3. Thyroiditis progression was characterized by an initial phase of hyperthyroidism followed by the development of hypothyroidism, consistent with painless thyroiditis. Patients with transient or subclinical TSH alterations (*n* ═ 3, 6%) were monitored closely but were not classified as having thyroid dysfunction unless they met the diagnostic criteria. TFTs were measured at baseline and every six weeks during ICI therapy, with additional testing performed if clinically indicated.

Management of hypothyroidism involved levothyroxine, typically starting at 25–50 µg/day, while hyperthyroidism was treated with beta-blockers and, when necessary, antithyroid medications. All treatment approaches adhered to the European Thyroid Association (ETA) recommendations [16].

### PD-L1 assessment

PD-L1 immunohistochemistry (IHC) was performed using either the 22C3 pharmDx assay (Dako) or the Ventana SP142/SP263 assays, following drug-specific protocols. For pembrolizumab, the Tumor Proportion Score (TPS) was assessed using the 22C3 pharmDx assay, while atezolizumab evaluation followed the manufacturer’s instructions for the Ventana SP142 or SP263 assays. In our analyses, PD-L1 expression was categorized as <1%, 1%–49%, or ≥50%, based on the TPS or the corresponding Ventana scoring criteria.

### Time-dependent covariate analysis for lead-time bias

To address potential lead-time (immortal time) bias—where patients who develop thyroid dysfunction may inherently have longer treatment exposure—we performed a time-dependent covariate analysis. In this approach, patients were classified in the “no dysfunction” group until the time of thyroid dysfunction diagnosis.

### Outcome measures

PFS was defined as the time from initiation of ICI therapy to either disease progression (as determined by RECIST 1.1 criteria) or death. OS was defined as the time from ICI initiation to death from any cause, with patients censored at the time of their last follow-up. Radiologic assessments were conducted every 12 weeks using computed tomography (CT) scans. Treatment responses were categorized as complete response (CR), partial response (PR), stable disease (SD), or progressive disease (PD).

### Data handling and missing data

Each patient with missing or incomplete data was reviewed individually. When appropriate, multiple imputation methods were applied, and sensitivity analyses were conducted to assess the robustness of the findings. Any discrepancies in the data were resolved by consensus among independent reviewers.

### Ethical statement

All procedures were conducted in accordance with the Declaration of Helsinki and institutional guidelines, ensuring patient confidentiality and data protection.

### Statistical analysis

All statistical analyses were performed using IBM SPSS Statistics version 23.0 (IBM Corp., Armonk, NY, USA). A two-sided *P* value of <0.05 was considered statistically significant. Descriptive statistics were used to summarize baseline variables. Group comparisons—such as between patients with and without thyroid dysfunction—were conducted using Student’s *t*-test or the Mann–Whitney *U* test for continuous variables, and the chi-square test or Fisher’s exact test for categorical variables. Survival analyses for PFS and OS were estimated using the Kaplan–Meier method, with differences between groups assessed via the log-rank test. Multivariate analysis using Cox proportional hazards models was performed to adjust for potential confounders (e.g., age, gender, ECOG performance status, PD-L1 expression), and the proportional hazards assumption was confirmed using Schoenfeld residuals. To minimize immortal time bias, a time-dependent covariate for thyroid dysfunction was introduced.

## Results

### Patient characteristics

A total of 50 patients with metastatic NSCLC and an ECOG performance status of two or lower met the inclusion criteria. The median follow-up duration was 10.6 months (IQR: 4.1 months). By the time of data cutoff, nine patients (18%) had died. Table 1 summarizes the baseline characteristics: the median age was 68 years (range: 59–82), with 27 male (54%) and 23 female (46%) patients. The cohort included both adenocarcinoma and squamous-cell carcinoma histological subtypes. TNM staging was documented for all patients, with the majority (86%) diagnosed with stage IV disease. Tumor grade information was available for 22 individuals; of these, 14 were classified as G2 and 8 as G3.

**Table 1:** Baseline characteristics of patients

**Characteristic**	**Total** **(*n* ═ 50)**	**With thyroid dysfunction** **(*n* ═ 24)**	**Without thyroid dysfunction** **(*n* ═ 26)**	**P value
Age (years), median (range)	68 (59–82)	70 (59–76)	64.5 (60–82)	0.06
Gender, *n* (%)				0.98
Male	27 (54%)	13 (54.2%)	14 (53.8%)	–
Female	23 (46%)	11 (45.8%)	12 (46.2%)	–
ECOG performance status, *n* (%)				0.74
0	32 (64%)	16 (66.7%)	16 (61.5%)	–
1	14 (28%)	7 (29.2%)	7 (26.9%)	–
2	4 (8%)	1 (4.2%)	3 (11.5%)	–
TNM stage, *n* (%)*				–
IIIB/III C	7 (14%)	4 (16.7%)	3 (11.5%)	–
IV	43 (86%)	20 (83.3%)	23 (88.5%)	–
Tumor grade (subset *n* ═ 22)**				–
G2	14 (64%)ˆ	9 (75.0%)†	5 (50.0%)‡	–
G3	8 (36%)ˆ	3 (25.0%)†	5 (50.0%)‡	–
PD-L1 expression, *n* (%)				0.19
<1%	11 (22%)	4 (16.7%)	7 (26.9%)	–
1%–49%	16 (32%)	7 (29.2%)	9 (34.6%)	–
≥50%	23 (46%)	13 (54.2%)	10 (38.5%)	–

*Most patients presented with advanced disease (stage IV). ***Tumor grade data were available for 22 patients only. ˆPercentage of the 22 total patients with known grade (G2 ═ 14/22, G3 ═ 8/22). †Percentage of the 12 patients (within the 24 “With Dysfunction”) who had known grade. ‡Percentage of the 10 patients (within the 26 “Without Dysfunction”) who had known grade. Abbreviation: PD-L1: Programmed death-ligand 1.

### Treatment regimens

Regarding treatment regimens, 13 patients (26%) received pembrolizumab monotherapy for tumors with PD-L1 expression ≥50%, while 37 patients (74%) were treated with atezolizumab, either as monotherapy or in combination with chemotherapy. The median number of ICI therapy cycles administered was 19.5 (IQR: 19).

### Incidence of thyroid dysfunction

Thyroid dysfunction was observed in 24 of the 50 patients (48%), manifesting as hypothyroidism (12 patients, 24%), hyperthyroidism (four patients, 8%), or a hyperthyroid-to-hypothyroid transition consistent with the progression of thyroiditis (eight patients, 16%). The median time to onset for hypothyroidism was 10 treatment cycles (IQR: 5), while hyperthyroidism typically appeared earlier, at a median of six cycles (IQR: 19). Additionally, three patients (6%) experienced transient or subclinical TSH alterations that normalized without pharmacological intervention.

### Association with treatment type

Among the 24 patients who developed thyroid dysfunction, 21 (87.5%) were receiving atezolizumab and 3 (12.5%) were on pembrolizumab. Thyroid dysfunction occurred significantly more frequently in patients treated with atezolizumab than in those treated with pembrolizumab (*P* ═ 0.04).

### Association with demographic and clinical factors

No significant association was found between the development of thyroid dysfunction and either patient age (68.8 ± 4.5 vs 66.6 ± 5.7 years; *P* ═ 0.06) or gender (males: 13/27 vs females: 11/23; *P* ═ 0.98). While PD-L1 expression did not differ significantly between groups when treated as a continuous variable, categorically, the majority of patients in both groups had PD-L1 ≥1%.

### Management of thyroid dysfunction

Of the 24 patients who developed thyroid dysfunction, 16 (66.7%) required pharmacological management. All 12 patients with hypothyroidism were treated with levothyroxine. Among those who experienced transient hyperthyroidism, four were managed with beta-blockers alone, with no need for antithyroid drugs. Patients who transitioned from hyperthyroidism to hypothyroidism were closely monitored and started on levothyroxine therapy as needed.

### Progression-free survival

**Figure 1.**
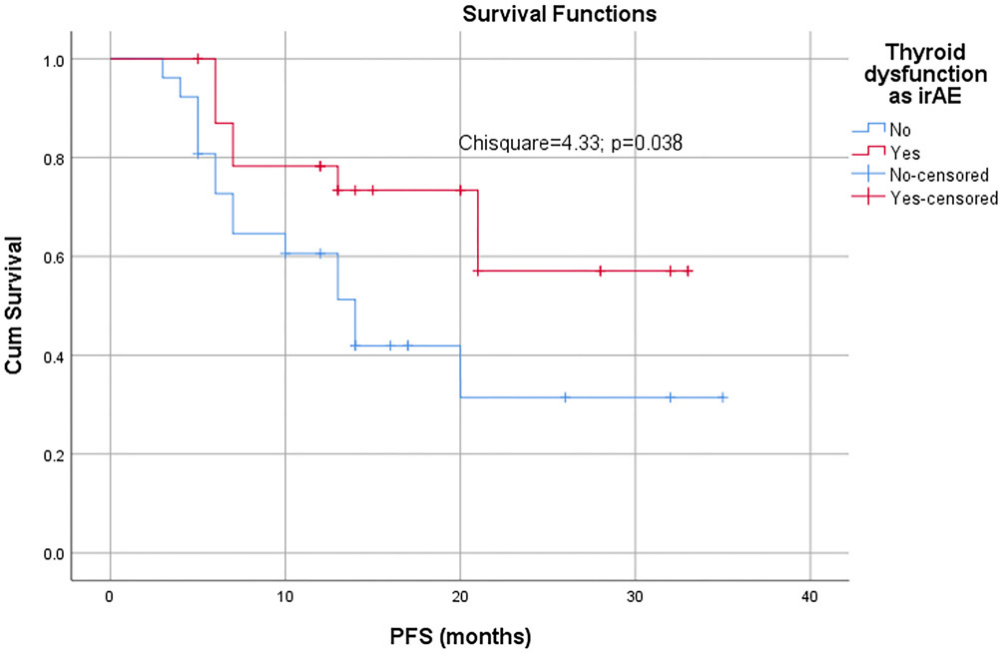
**Kaplan–Meier curve for progression-free survival.** The red line represents patients with thyroid dysfunction; the blue line represents those without thyroid dysfunction. A statistically significant difference in PFS is observed (*P* ═ 0.038, log-rank test), with patients who develop thyroid dysfunction showing improved outcomes. Abbreviations: irAE: Immune-related adverse event; PFS: Progression-free survival.

At a median follow-up of 10.6 months, the overall cohort had a median PFS of 21 months (95% CI: 11.6–30.4). Patients who developed thyroid dysfunction had a significantly longer PFS (median not reached) compared to those without dysfunction (14 months; 95% CI: 9.68–18.32; *P* ═ 0.038 by log-rank test; Figure 1). This association remained significant in a time-dependent covariate analysis that accounted for the variable onset of thyroid dysfunction; patients who developed dysfunction continued to demonstrate improved PFS compared to those who did not.

Although the median follow-up for the entire cohort was 10.6 months (IQR: 4.1), the Kaplan–Meier–estimated median PFS was 21 months (95% CI: 11.6–30.4). This is not contradictory, as time-to-event analyses reflect only those patients who either experienced progression or had sufficient long-term follow-up by the analysis cutoff. Some participants enrolled early and remained progression-free for over two years, thus influencing the estimated median PFS. In contrast, those enrolled later had shorter follow-up durations and were censored if they had not yet progressed.

### Overal survival

OS data were immature at the time of this analysis, with the median OS not reached in either group. Further follow-up is needed to determine whether the PFS benefit observed in patients with thyroid dysfunction translates into an OS advantage.

### Multivariate analysis

A Cox proportional hazards model, adjusted for age, gender, ECOG performance status, PD-L1 expression (categorized), and the presence of other irAEs, identified PD-L1 expression as the only independent predictor of improved PFS (HR: 0.98, 95% CI: 0.969–0.997, *P* ═ 0.02). Neither age, gender, nor the presence of thyroid dysfunction remained independently significant after adjusting for PD-L1, although thyroid dysfunction showed a trend toward a favorable hazard ratio.

### Subgroup analysis

In the subgroup analysis, among the 37 patients treated with atezolizumab, those who developed thyroid dysfunction had a significantly longer median PFS compared to those who did not (not reached vs 13 months; *P* ═ 0.006). In contrast, only three of the 13 patients in the pembrolizumab cohort developed thyroid dysfunction, limiting the statistical power for a meaningful comparison.

### Safety and tolerability

Overall, ICI therapy was well tolerated. Grade ≥3 adverse events occurred in three patients (6%) but were not thyroid-related. No patients discontinued ICI therapy due to thyroid dysfunction.

## Discussion

In this retrospective cohort study, we examined the incidence and clinical impact of thyroid dysfunction among patients with metastatic NSCLC receiving ICI therapy at our institution. Thyroid dysfunction occurred in nearly half of the patients (48%), aligning with the higher-end estimates reported in the literature [11, 17]. Notably, most patients who experienced thyroid dysfunction were receiving atezolizumab, suggesting that PD-L1 blockade may carry a higher risk of thyroid irAEs than PD-1 blockade, as has been observed in other studies [11, 18].

An important finding was the association between thyroid dysfunction and prolonged PFS, a relationship previously suggested in other studies [14, 15, 19]. Although the exact biological mechanisms remain unclear, it is hypothesized that the same heightened immune response responsible for thyroid autoimmunity may also contribute to enhanced anti-tumor activity [20, 21]. Our time-dependent covariate analysis further supported this association by minimizing lead-time bias, suggesting that the timing of thyroid dysfunction during therapy is relevant to patient outcomes. It is worth noting that, although the median PFS is 21 months, this estimate is based on a subset of patients with sufficient follow-up (primarily early enrollees). Ongoing follow-up will help refine this estimate, and the current 95% CI of 11.6–30.4 months reflects the limited number of events observed at later time points.

We did not observe a direct relationship between the severity of thyroid dysfunction—as indicated by TSH, FT4, or FT3 levels—and clinical benefit; rather, it was the presence of dysfunction itself that appeared to be relevant. This finding is consistent with reports suggesting that even subclinical or mild thyroid abnormalities may reflect broader immune activation [22]. No significant associations were found with age or gender, indicating that all patients receiving ICIs, regardless of demographic factors, require careful thyroid monitoring.

Only approximately 30% of patients with advanced cancer benefit from ICI therapy [23], underscoring the need for more effective predictive biomarkers to guide treatment decisions. Tumor PD-L1 expression, assessed by IHC, is currently the most commonly used criterion for patient selection; however, its predictive power is limited when used alone [23]. Emerging evidence suggests that both baseline values and dynamic changes in systemic inflammatory and nutritional indices—such as the HALP (hemoglobin, albumin, lymphocyte, and platelet) score, as well as the neutrophil-to-lymphocyte ratio (NLR) and platelet-to-lymphocyte ratio (PLR)—are associated with immunotherapy efficacy and can reliably predict outcomes in advanced NSCLC [24]. Notably, patients with low HALP scores combined with high NLR or PLR values tend to have significantly poorer prognoses, indicating that these readily available markers may serve as valuable tools for stratifying patients in the context of immunotherapy [24].

Our study has several limitations. Its retrospective, single-center design limits generalizability and introduces potential selection bias. Although the sample size reflects our institution’s experience, it remains relatively small, particularly for subgroup analyses. Furthermore, we lacked comprehensive data on thyroid autoantibodies, which could have provided mechanistic insights into ICI-induced thyroiditis [25]. Lastly, OS data remain immature, and extended follow-up is needed to determine whether the observed PFS advantage translates into a survival benefit.

Future research should prioritize prospective, multicenter trials with larger cohorts that incorporate routine measurements of thyroid autoantibodies and other immunological markers (e.g., cytokine profiles). Investigating the impact of early detection and management of thyroid irAEs on clinical outcomes would also be valuable. Additionally, refining PD-L1 assessment—potentially in combination with other biomarkers—may help identify patients at higher risk of developing thyroid irAEs and those who may respond more favorably to ICI therapy [26].

Clinically, our findings highlight the importance of regular thyroid function testing in patients receiving ICIs, particularly atezolizumab. Early detection and management of thyroid dysfunction are essential to maintaining quality of life and avoiding treatment interruptions. Although not yet conclusive, the potential role of thyroid dysfunction as a biomarker of ICI response remains a promising area for further research [27].

## Conclusion

Thyroid dysfunction is a common irAE in patients with metastatic NSCLC receiving ICIs—particularly atezolizumab—and may be associated with improved PFS. These findings underscore the importance of systematic thyroid monitoring in this population and suggest that thyroid dysfunction could serve as a surrogate marker of enhanced immunotherapy efficacy. Further prospective research is warranted to elucidate the underlying mechanisms and validate the potential prognostic significance of this endocrine irAE.
